# Osteoblastoma of the Os Capitatum

**DOI:** 10.1155/2014/241716

**Published:** 2014-07-03

**Authors:** Çağrı Kaptan, Halil Atmaca

**Affiliations:** Department of Orthopaedics and Traumatology, School of Medicine, Akdeniz University, 07058 Antalya, Turkey

## Abstract

An osteoblastoma is a primary benign bone tumor, which is rarely seen in hand bones. Osteoblastoma is generally seen in spine, pelvis, and long bones. However, there are a few case reports of osteoblastoma in wrist and hand bones. To our knowledge, up to now, only one male patient with osteoblastoma in capitate bone was reported. We report the first female case of osteoblastoma of capitate bone and discuss diagnosis and treatment.

## 1. Introduction

Osteoblastoma is a primary bone tumor constituting 1% of all bone tumors [1]. It is usually seen in patients aged between 10 and 25 years [2]. This tumor is usually located in pelvis, vertebral columns, and long bones but rarely in hand and wrist [3]. Differential diagnoses include giant cell tumors, osteoid osteoma, chondrosarcoma, aneurismal bone cyst, and osteosarcoma [4].

Osteoblastoma is usually seen in men (men/women: 2/1) [1]. Up to now, only one male patient was reported with osteoblastoma of the capitate bone [5]. We describe the first female patient with an osteoblastoma of the capitate and discuss diagnostic steps, treatment approach, and prognosis.

## 2. Case Report

**Figure 1:**
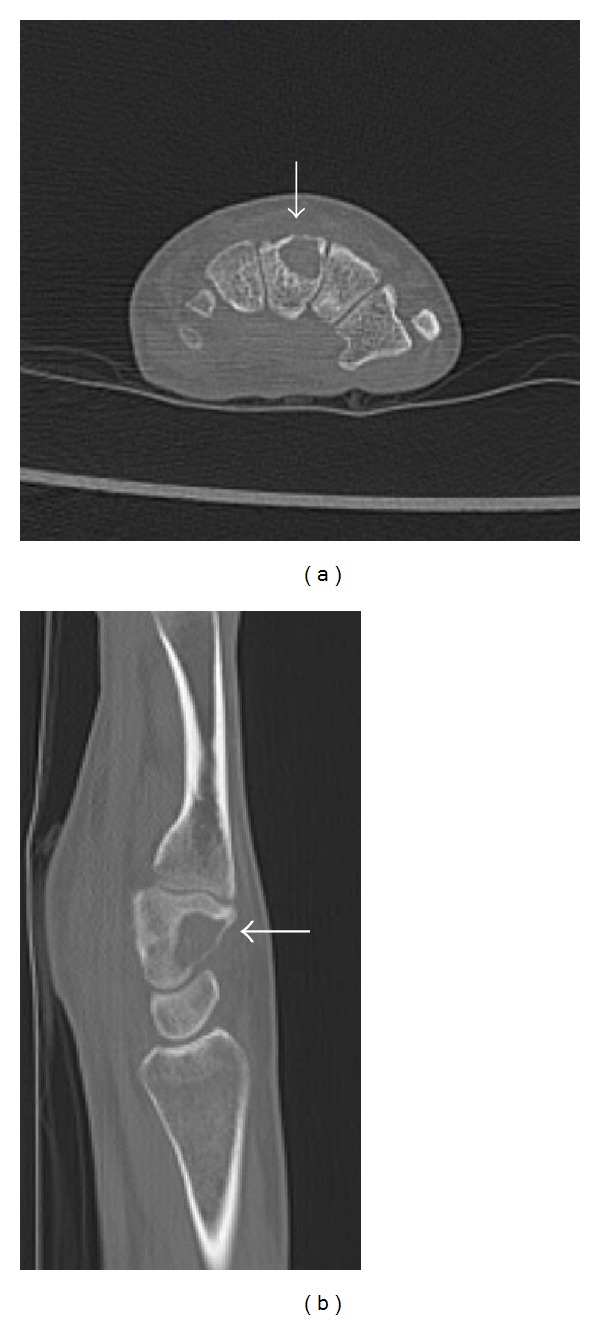
CT images of the patients. Cystic lesion with sclerotic margin is seen in axial plane and sagittal plane ((a) and (b), resp.). White arrows are showing the cystic lesion.

A right-handed 21-year old woman presented with a 7-month history of painful swelling of the dorsal middle area of the right wrist. She denied relief of the pain from aspirin and nonsteroidal anti-inflammatory drugs. There was no decrease in the range of motion of the wrist. No history of trauma of the hand or wrist was reported. Physical examination was remarkable for an immobile, firm, and painful swelling. Routine laboratory tests were in normal range. There was not an obvious abnormality in the anteroposterior plain radiographs of the right wrist. Additionally computed tomography (CT) scans showed a well-defined lesion located in the dorsal portion of capitate bone (Figure 1).

**Figure 2:**
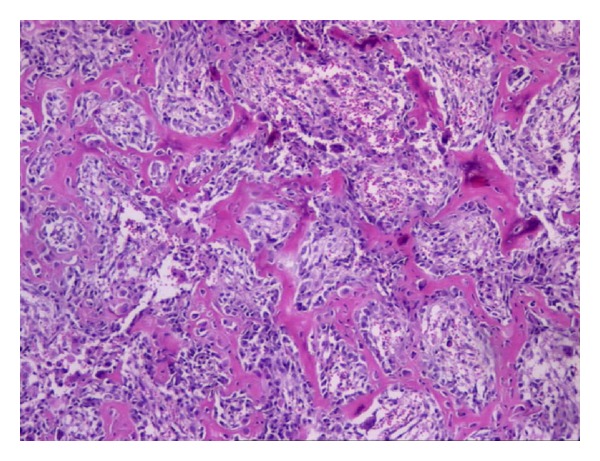
Sharply demarcated tumor composed of anastomosing bony trabeculae surrounded by a loose fibrovascular stroma. H&E ×100.

Differential diagnoses include osteoblastoma, giant cell tumor, osteoid osteoma, chondrosarcoma, aneurysmal bone cyst, and osteosarcoma. Percutaneous Trucut biopsy was performed by direct dorsal approach. The biopsy material was diagnosed as osteoblastoma. Therefore, the lesion was treated by curettage and a bone graft was used to fill the lesion. Pathological results revealed an osteoblastoma of the capitate bone (Figure 2).

A rapid improvement in pain was observed after the curettage biopsy. The patient did not need to use any pain killers after the first week of the operation. There was no change in the range of motion of the right wrist. During the 15-month follow-up, the patient reported no abnormalities. The patient gave her informed consent to our treatment method and publication of this report. She continues to be followed up.

## 3. Discussion

Osteoblastoma is generally seen in spine, pelvis, and long bones. However, there are a few case reports of osteoblastoma in wrist and hand bones such as scaphoid [6, 7], hamatum [8, 9], and triquetrum [10]. To our knowledge, up to now, only one patient with osteoblastoma in capitate bone was reported [5]. In that report, the patient was male and the lesion was secondary to trauma. Although osteoblastoma is generally seen in males [1], our patient was a female and she denied any history of trauma. Additionally, the patient of Afshar [5] had lost the wrist range of motion. However, our patient had no change in the range of motion of the wrist.

Osteoid osteoma should be considered in the differential diagnosis of osteoblastoma because they have similar clinical findings. The size of the lesion is one of the most important differences between osteoid osteoma and osteoblastoma, the former being smaller than 2 cm and the latter being greater than 2 cm [11]. In our case, however, the lesion was smaller than 2 cm; hence the diagnosis was not based on the size of the lesion. Histopathologically, osteoblastoma is characterized as abundant and vascularized loose connective tissue [9].

We believe our case is the second report of osteoblastoma in capitatum. Although osteoblastoma of the capitatum is rare and diagnosis is not simple because of the nonspecific clinical and radiological findings, histopathology may help making the diagnosis. There are different kinds of surgical treatments such as curettage, curettage and graft, and proximal row carpectomy [9]. Although this report mainly suffers from short-term follow-up, only curettage and bone graft may provide to achieve good functional results without recurrence.
